# A Web-Based Intervention for Youth With Physical Disabilities: Comparing the Role of Mentors in 12- and 4-Week Formats

**DOI:** 10.2196/15813

**Published:** 2020-01-08

**Authors:** Sally Lindsay, Elaine Cagliostro

**Affiliations:** 1 Bloorview Research Institute Holland Bloorview Kids Rehabilitation Hospital Toronto, ON Canada; 2 University of Toronto Toronto, ON Canada

**Keywords:** social support, mentor, youth, adolescent, employment

## Abstract

**Background:**

Youths with physical disabilities face many barriers in society, including social exclusion, stigma, and difficulties finding employment. Electronic mentoring (e-mentoring) offers a promising opportunity for youths with disabilities and has the potential to improve their inclusion while enhancing career outcomes. However, little is known about the role of mentors in a Web-based e-mentoring format to improve employment outcomes.

**Objective:**

This study aimed to explore the role of mentors in engaging youths in an e-mentoring intervention and to compare and contrast mentors’ engagement strategies within a 12- and 4-week format.

**Methods:**

This paper drew on a pilot feasibility study, which is a group, Web-based employment readiness intervention involving a discussion forum for youths with physical disabilities. Our intervention involved having trained youth mentors (ie, near-peers who also had a disability) lead Web-based discussion forums while offering peer support and resources, which involved 12 modules completed over both a 12- or 4-week format. We used a mixed method approach including qualitative data (mentor interviews and discussion forum data) and quantitative data (pre-post survey data) comparison.

**Results:**

A total of 24 youths participated across 3 e-mentoring intervention groups: 9 in the 12-week format (mean age 17.7 years [SD 1.7]) and 15 in the 4-week format (mean age 19.5 years [SD 2.6]), led by 3 trained youth mentors with disabilities, 2 males and 1 female (mean age 22 years [SD 2.64]). Our findings revealed that mentors engaged youths in the e-mentoring program by providing informational, emotional, and tangible support. We noted more instances of mentors providing advice, empathy, and encouragement in the 12-week format compared with the 4-week format. We also found fewer examples of providing advice, developing a rapport, and social support from mentors in the 4-week format. Our findings revealed no significant differences between the 2 groups regarding time spent in the forum, number of logins, number of posts, and self-rated engagement.

**Conclusions:**

Mentors in the 12-week and 4-week format engaged participants differently in providing informational and emotional support, although there were no differences in tangible support provided. Mentors reported that the 12-week format was too long and lacked interaction between participants, whereas the 4-week format felt rushed and had fewer detailed responses from mentees.

**International Registered Report Identifier (IRRID):**

RR2-10.2196/resprot.8034

## Introduction

### Background

Youths with disabilities are at risk of living below the poverty line and having poor developmental outcomes [1-5]. They often experience social exclusion and isolation and encounter challenges of being fully included within society. The hurdles that youths encounter are often a result of their social (eg, stigma and discrimination) and physical (eg, inaccessible spaces) environments [6,7]. Approximately 4.4% of youths aged 15 to 24 years in Canada have a disability [8] (defined as impairments, activity limitations, and participation restrictions) [9], and 1% of these youths have a physical disability. Therefore, it is critical to find ways to enhance their inclusion. Focusing on youths with physical disabilities is important because they are considered a vulnerable population with unique social and vocational needs [10]. Youths with physical disabilities often experience different challenges than youths with chronic illnesses, because their condition is often visible and they may face difficulties with mobility, speech, stigma, coping, and social exclusion [6,10,11]. Furthermore, they often experience periods of developmental, emotional, and social changes, which differ from other youths [12-14].

One encouraging approach to improving the inclusion of underrepresented groups, such as youths with disabilities, is through peer mentoring [15]. A mentor is someone who acts as a role model and shares experiences while supporting a protégé in their development [16-18]. Peer mentoring refers to those of similar age who share experiential knowledge and lived experiences as a mechanism for promoting positive outcomes [19]. Peer mentors can offer tangible, informational, and emotional support for youths [20,21]. Mentors typically perform 3 main functions: vocational or instrumental support, psychological support through counseling, friendship, and encouragement [22-26]. Such traits are often linked with positive mentor-related outcomes [27-29]. For example, among youths without disabilities, there is a strong empirical basis showing the effectiveness of mentoring on improved self-efficacy, quality of life, and employment [17,24,30-32]. Mentoring targeted toward a specific achievement or goal is referred to as instrumental mentoring [33]. For instance, mentoring is an important component of career development [34], whereby those who are mentored have better opportunities for advancement, make higher salaries, and report higher career satisfaction [35].

Research on youths with disabilities indicates that mentoring can have beneficial impacts on the development of educational, employment, social skills and on self-esteem [2,30,36-38]. A recent systematic review on disability and mentoring found that mentoring led to improvements in knowledge of employment services and support [39,40] and knowledge of employment preparation [39-41] and employment outcomes [42]. For example, Kolakowsky-Hayner et al [43] had a group-based mentoring program to help youths with acquired brain or spinal cord injury to return to work and school. They found their program helped the youths achieve educational goals [43].

Most studies in this area focus on traditional face-to-face mentoring in a one-to-one or group-based format [17]. Despite the potential benefits of mentoring for youths with disabilities, they may encounter challenges in accessing mentors in person because of geographical or other barriers and limited mobility [17,44]. Therefore, Web-based formats may offer a viable alternative, helping to address some of these limitations.

Electronic mentoring (e-mentoring; ie, computer mediated) is a newer format of mentoring [45] helping to meet the needs of underserved populations, such as youths with disabilities [17,46,47]. Mentoring models have evolved considerably since the original face-to-face relationships [48] toward Web-based relations that are sustained primarily through electronic means [45] and multiple mentoring with 1 protégé having multiple sequential mentoring relations [49].

E-mentoring offers opportunities that are not seen in face-to-face mentoring, such as creating written text, practicing communication skills, and developing relationships without barriers of time and distance [22], along with immediate access to mentors [50]. There is also a chance to be mentored by several people from varying backgrounds [34]. Furthermore, the anonymity of electronic formats can provide a degree of privacy that is not always possible with face-to-face mentors [34]. E-mentoring relationships have the potential to generate a sense of belonging and autonomy, satisfaction, fulfillment, and potential friendships [51]. Some studies highlight that electronic mentors (e-mentors) can help to enhance employment and career-related outcomes. For example, in a Web-based mentoring program for youths with vision impairments, Bell [36] found a significant increase in efficacy to make career-related decisions compared with the beginning of the program. Similarly, Kim-Rupnow and Burgstahler [41] had a community Web-based mentoring program for youths with disabilities and found a significant improvement in knowledge of career options. Other research on mentoring for youths with disabilities to enhance employment or academic outcomes has used face-to-face formats [17,39,42,43]. Little is known about the role of mentors in a Web-based format aiming to improve employment skills.

### Rationale

This study addressed several important gaps in the literature. First, there is an underrepresentation of youths with disabilities in mentoring [17], especially where mentors have a disability themselves. Most studies focus on mentors without disabilities. Having youths with a disability who mentor other youths may be valuable, given they have shared lived experiences [15,52]. Second, little is known about the role of e-mentoring for youths with physical disabilities, especially in the context of employment preparation. Third, our study is novel because it compares 2 different lengths (ie, 12 and 4 weeks) of the same mentoring intervention (see studies by Lindsay et al [53-55] for the full description).

## Methods

### Objectives

The objectives of this study were (1) to explore the role of mentors in engaging youths in an e-mentoring employment preparation intervention for youths with physical disabilities and (2) to compare and contrast mentors’ experience and engagement strategies and level of engagement within a 12- and 4-week format.

### Design

This paper drew on a feasibility, embedded, qualitative pilot randomized controlled trial design [56], assessing a group, Web-based employment readiness intervention (*Empowering youth towards employment*) involving a discussion forum for youths with physical disabilities [10]. This intervention included (1) experimental groups receiving employment preparation Web-based modules and a peer e-mentor and (2) control groups receiving the Web modules only (with no mentor) but could interact with other participants within their group [10]. The intervention consisted of 12 modules on employment preparation [10]. The discussion forums were hosted on a youth- and disability-friendly website (*Ability Online*, by using Web-hosting *Drupal* platform analytics) through a unique link that only participants could access (for a full description, see the study by Lindsay et al [10]). For this paper, we focused only on the 3 experimental groups that received the intervention with 2 youth mentors (see Multimedia Appendix 1).

### 12- Versus 4-Week Format

From the 3 experimental groups, the first group ran for 12 weeks, whereas the following 2 groups were condensed to a 4-week format, which we adjusted based on participant feedback [53]. Group 1 included a 12-week long program, which consisted of 1 topic per week. Participants in the 4-week intervention (groups 2 and 3) received the same topics and information, but 3 topics were posted per week for 4 weeks. Mentors divided the discussion topics in half between them, with each mentor posting 6 of the 12 topics, which allowed participants to get to know both mentors equally (see Multimedia Appendix 1).

### Mentor Training

All mentors completed mentor training (ie, hospital-based youth peer mentor training program and project-specific training), received research ethics training, and had employment experience at the time of the intervention. Mentors were trained on how to use the *Ability Online* platform, and they had to introduce the topics in the same order. They were trained to respond to participant’s comments in a similar manner, providing information, appraisal, and emotional support (eg, active listening, perspective taking, maintaining boundaries, positive modeling, trust building, interactive training, and mentoring) [10]. A research coordinator and project director supervised the mentors.

### Procedures

We received institutional research ethics board approval. Eligible participants received an information letter and a phone call from the researchers who screened all participants and obtained informed written consent before enrolling them in the intervention. Once participants consented, they were randomized into an experimental or control group of up to 10 participants in each group [10]. Following that, a researcher contacted participants to inform them of their group assignment and instruct them on the procedures to be followed, including a presurvey and registering for the Web-based discussion forum.

### Recruitment and Participants

Participants were recruited in the summer months from June 2016 to August 2018 through invitation letters sent from a pediatric hospital and advertisements. Inclusion criteria were as follows: (1) able to read and write in English, (2) aged 15 to 25 years, (3) have access to a computing device with internet access; (4) currently enrolled in or recently completed a high school diploma in the applied or academic stream (ie, university- or college-bound students), (5) have no paid work experience, and (6) youths with a physical disability [10]. Youths who were thought to meet the inclusion criteria were sent an invitation letter from our hospital database. Our rationale for this age group and for choosing youths without employment experience is that youths with disabilities often start their first job later than youths without disabilities [10]. Exclusion criteria involved those who recently completed or who are currently participating in another employment preparation or peer support intervention.

### Data Collection

#### Mentor Interview Data

A researcher conducted semistructured interviews with each mentor after the completion of forums for each group (from October 2016 to September 2018). There were 6 interviews conducted in total. Questions asked were about what mentors liked most and least from the intervention, how engaged the participants were, and how engaged they were in the group (for interview guide, see Multimedia Appendix 2).

#### Discussion Forum Data

We drew on the discussion forums for each group, which consisted of 12 topics. Mentors posted an introduction to each topic with information and examples from their personal experiences and a series of discussion questions for the mentees to respond to. Mentees replied to the discussion questions and shared their experiences, which mentors replied to individually. We also analyzed quantitative data from the discussion forums, including the number of logins, number of posts per participant, and total time logged in to the discussion forum.

#### Pre-Post Survey Data

We drew on quantitative data from the pre-post surveys to compare and contrast differences between those in the 4-week and 12-week groups. Surveys were sent out to all participants via email. All 25 participants completed the presurvey, and 19 participants completed the postsurvey (9 in group 1, 5 in group 2, and 5 in group 3). Of 25 participants, 6 did not complete the survey either because of losing interest in the program or because they could not be reached by the research team for follow-up. We analyzed the following 2 variables: self-rated level of engagement and whether participants would recommend the program to others.

### Data Analysis

#### Mentor Interviews

Mentor interview data were audio recorded, transcribed verbatim, and anonymized and checked for accuracy. The semistructured interviews with mentors were analyzed using a qualitative thematic analysis [57]. With the research question in mind, the interview transcripts were analyzed by both authors independently. Then, a sample transcript was independently read and coded by each individual. Codes were categorized by types of social support given (eg, informational, emotional, and tangible). Codes were collated into larger categories (ie, themes). After this, we met to compare and contrast codes and arrived at consensus to create a final codebook, which were applied to all transcripts using NVivo. We kept an audit trail, documenting all decisions and discrepancies noted throughout the coding and analysis process. The codebook was then used to further analyze transcripts and extract quotes representative of the themes and subthemes of the results.

#### Discussion Forum Data Analysis

We downloaded the discussion forum data from the host website, stored as a password-protected document, and entered them into NVivo 10 and analyzed them using qualitative thematic analysis. We chose this approach because of its flexibility to analyze a variety of data types and sample sizes [58]. This method is useful when analyzing semistructured interviews and large discussion forum data, where we had 24 unique participants, 3 mentors, and 162 pages of data. We organized and coded the initial dataset using an open coding, iterative approach, which was informed by our research question. Both authors read a sample of the discussion forum transcripts and coded them independently and later met to discuss codes until we reached a final consensus with the coding scheme. The codes were then applied to the entirety of the dataset, where they were categorized into themes and subthemes.

#### Qualitative Comparison

After forum transcripts and interviews were coded once in entirety, they were compared and contrasted again using a constant qualitative comparative method to analyze differences within and between the 12- and 4-week intervention groups [59]. We developed a thematic comparison table to help analyze what themes were present in each comparison group (see Multimedia Appendix 3), and representative quotes were abstracted of the themes analyzed within and across groups.

#### Survey Data

Data from the surveys were analyzed using descriptive statistics and *t* tests by using SPSS version 25 to explore differences between the 12- and 4-week format. Forum usage was tracked using the Drupal software built into the Web hosting platform (eg, time spent in the forum was measured as total overall time on the website in hours, number of log-ins, and number of posts). Self-rated engagement was measured on a 10-point scale (1=low engagement and 10=high engagement). Recommendation of the program to others was a dichotomous variable (1=yes and 0=no).

## Results

### Sample Characteristics

We first outlined the sample characteristics followed by differences between groups with regard to time spent in the forum, number of posts, and self-rated engagement. Then, we explored how mentors engaged youths within the discussion forum.

Our sample consisted of 27 participants: 24 mentees and 3 youth mentors. We had 9 participants in the 12-week format (mean age 17.7 years; 5/9, 55% females) and 15 participants in the 4-week format (mean age 19.5 years, 9/15, 60% females). They had various physical disabilities, including cerebral palsy, muscular dystrophy, Charcot-Marie tooth disease, and spina bifida (see Table 1).

**Table 1:** Mentee and mentor demographics.

Demographics		Participants		Mentors (n=3)
		12 weeks (n=9)	4 weeks (n=15)	
Age (years), mean (SD)		17.7 (1.7)	19.5 (2.6)	22 (2.64)
**Sex, n (%)**				
	Male	4 (44)	6 (40)	2 (67)
	Female	5 (56)	9 (60)	1 (33)
**Disability type, n (%)**				
	Cerebral palsy	4 (44)	7 (47)	2 (67)
	Duchenne muscular dystrophy and neuromuscular	5 (56)	3 (20)	0 (0)
	Spina bifida	0 (0)	2 (13)	0 (0)
	Other physical disabilities	0 (0)	3 (20)	1 (33)
**Group, n (%)**				
	1	9 (100)	0 (0)	2 (67)
	2	0 (0)	7 (47)	2 (67)
	3	0 (0)	8 (53)	2 (67)

Mentors included 2 males and 1 female with a disability, aged between 19 and 25 years. Each mentored group had 2 mentors (1 male and 1 female), who alternated posting topics. Mentor 1 was a female, aged 20 years, who was enrolled in postsecondary education at the time of the intervention and participated in all 3 groups. Mentor 2 was a male, aged 19 years, who was enrolled in postsecondary education, who mentored groups 1 and 2, but was unable to continue on as a mentor for the last group. Therefore, mentor 3 was introduced in group 3 and included a male, aged 25 years, who completed postsecondary education.

### How Mentors Engaged Youths Within the Discussion Forum

Throughout the e-mentoring intervention, youth mentors used several strategies to engage participants and encourage interaction within the discussion forums. We explored strategies that mentors commonly used (ie, informational, emotional, and tangible support) and to what extent they varied between the 12- and 4-week formats.

Our findings showed the type of informational support in the 12-week format (ie, employment, postsecondary, and volunteering) included a greater breadth of topics than the 4-week format (ie, employment; see Multimedia Appendix 3). There were more examples of providing advice in the 12-week group compared with the 4-week format. Meanwhile, emotional support in the 12-week format involved more examples of empathy and understanding, whereas the 4-week format involved offering encouragement. There were no differences in the types of tangible support provided to participants across the 2 formats. Finally, we have outlined the differences in the mentor’s experience in the 12- and 4-week groups.

#### Informational Support

Mentors provided informational support, which included providing resources for employment-related issues, offering advice, and researching a specific topic for mentees (see Multimedia Appendix 3). All mentors provided informational support in both the 12- and 4-week format; however, the content differed between groups, with mentors providing additional support on employment, postsecondary education, transportation, and volunteering in the 12-week intervention group, whereas only providing additional employment-related support in the 4-week intervention group. We found more instances of providing advice and longer posts in the 12-week format compared with the 4-week format.

In the 4-week format, mentors provided informational support to youths in the form of employment tips and resources, in response to mostly work-related questions and concerns from youths. Although all youth mentors provided informational support, mentors in the 12-week format provided support on multiple topics, whereas the informational support in the 4-week format focused mainly on employment-related topics.

When comparing how mentors provided advice in both the 12- and 4-week formats, mentors gave longer and more detailed advice to mentees in the 12-week format (longer and with more information) compared with the 4-week format. Alternatively, advice given in the 4-week format typically included 1 or 2 sentences and contained less information. The discrepancy in advice given between groups was mentioned in a postintervention interview, where mentor 1 shared why she did not need to provide as much information in the 4-week format:

Mentees were saying a lot of the right things; So, I really didn't have too much else to add, other than one girl who didn't know what networking was; So, I explained that to her. I felt like it wasn't really too in-depth, because this group seemed like they knew what they wanted.

Mentor 1, Group 3

Our results indicate that the types of social support and advice shared by mentees differed between the 12- and 4-week formats. The ways in which it differed included more types of information shared with mentees in the 12-week group (ie, employment, postsecondary, and volunteering) and more instances of providing advice to mentees in the 12-week intervention group. Meanwhile, only employment-related information was shared in the 4-week format, and posts providing advice were shorter.

#### Emotional Support

Mentors provided emotional support, which involved encouragement, being vulnerable, and showing empathy to mentees. We noticed differences in the type of emotional support provided by mentors in the 12- and 4-week format. For example, mentors in the 12-week group provided more empathetic and understanding support to mentees while displaying vulnerability. Meanwhile, emotional support in the 4-week group comprised encouragement to mentees and focusing on solutions. Another difference included that although all 3 mentors provided emotional support, in both the 12-week and 4-week format, the female (mentor 1) provided the most variety and instances of emotional support to all mentees.

The instances of emotional support observed in the 4-week intervention group used encouragement rather than empathy. For example, mentor 3 stated:

That's amazing! You've really molded yourself some amazing experiences that will help your future goals. These are all amazing things to put on your resume.

Mentor 3, Group 3, 4-week format

The emotional support in the 4-week format reinforced the youth’s accomplishments and encouraged them to not give up on their goals. Another type of emotional support in the 12-week format included mentors showing vulnerability with mentees. In the 4-week format, mentors shared their challenges but focused on sharing solutions with mentees.

Mentors provided emotional support in the discussion forums to engage participants, although the methods differed in the 12- and 4-week formats, such as showing empathy, understanding, and vulnerability in the 12-week format and offering encouragement and solutions in the 4-week intervention groups. It is important to note that the female mentor offered more emotional support to mentees (of both genders) than male mentors.

#### Tangible Support

Mentors provided tangible support, which included offering solutions and additional support. One strategy involved mentors offering an alternative solution to mentees when they may not agree with them (see Multimedia Appendix 3). These occurrences were infrequent but appeared in both the 12-week and 4-week format. Mentor 1 used this strategy only in the 4-week format, whereas mentor 2 provided alternative solutions in both the 12- and 4-week format, and mentor 3 did not use this strategy at all. In particular, on the topic of managing one’s disability in the workplace, a male mentee in group 2 discussed their opinion of *keeping it to themselves* if they were discriminated against in the workplace. Mentor 2 responded:

I agree with most of what you said [but] I’m not sure I would handle discrimination in the workplace in the same way...Hopefully, you will never run into these situations, but not reporting them could have negative long-term effects.

Mentor 2, Group 2, 4-week format

In this instance, the mentor described that the situation could potentially result negatively and offered an alternative solution to the mentee on how to deal with it.

Another way mentors provided tangible support (in both the 12- and 4-week format) included offering additional help or support to mentees either through a follow-up post or private message. For instance, mentor 1 commonly used this technique. Mentor 2 offered additional support to mentees on occasion. For example:

If you have a question you would like to be answered in private or if you would just like to chat, feel free to send Mentor 1 or message.

Mentor 2, Group 2, 4-week format

We found no differences in tangible support provided within the 12- and 4-week intervention groups. Tangible support included offering solutions and additional help to mentees, although there were inconsistencies in using this strategy between mentors.

### Mentor Experience

#### Lack of Participation and Engagement

All mentors experienced challenges with engaging participants and shared disappointment in their expectation of the level of engagement (see Multimedia Appendix 4). This theme was evident in the 4-week postintervention interviews and briefly mentioned by mentors in the 12-week program. For example, Mentor 2 expressed:

I didn't really get to talk to (participants) as much as I thought I would originally because you encourage people to private message you and reach out, but most of them didn’t.

Mentor 2, Group 1

In the 4-week intervention groups, mentors expressed more concern over a lack of engagement despite their efforts. For example, Mentor 3 noted:

There was definitely a lack of participation. I tried to facilitate as much conversation back and forth as I could but it was still a lot of one note answers, and I just don’t think there was much across the board engagement.

Mentor 3, Group 3

Another concern with the participation in the 4-week intervention group involved the length and detail of posts from the mentees. Mentor 1 shared:

The way mentees were answering questions was a bit concerning because they were very quick and wouldn’t elaborate…I would ask them, what’s difficult about finding work? They would say: well my disability has limited me but they wouldn't elaborate.

Mentor 1, Group 2

Overall, mentors perceived lower than expected mentee engagement, but particularly in the 4-week intervention groups, where mentees’ posts were brief and less detailed.

#### Length of the Intervention

The length of the program was another issue that mentors reported affected participation and the quality of mentee posts. In the 12-week format (ie, 1 topic a week), mentors expressed concerns with the program being too long for participants, stating:

I would see a few people participate less as we progressed through the week, so they weren't able to get as much out of the program as I would have liked them to because it was such a long commitment.

Mentor 1, Group 1

Mentors discussed how the 12-week format affected their own engagement. To illustrate:

My motivations started to dip a tiny bit near the 11 and 12th week just because I wasn’t getting as much feedback from the participants as (I did) initially.

Mentor 1, Group 1

On the basis of feedback from the 12-week intervention group, we amended it to a 4-week format with 3 topics a week. One of the benefits of the 4-week program included:

It’s a shorter time commitment…and you still get the same amount of material but in a shorter amount of time.

Mentor 1, Group 2

Some criticisms of the 4-week program length included not having enough time to elaborate on topics and less interaction.

Mentors highlighted the benefits and limitations of the 12- and 4-week intervention, with an overall consensus that the 12-week format was prone to drop off in participant engagement but had more in-depth responses. Meanwhile, the 4-week format involved a more efficient time commitment for mentees yet felt rushed for mentors and did not allow time for meaningful interactions.

#### Levels of Engagement

Drawing on our pre- and postsurvey, we found no significant differences between the 2 groups regarding time spent in the Web forum (in the intervention), number of log-ins, number of posts, self-rated engagement, or whether they would recommend the program to others (see Table 2). Despite the lack of significance, there were notable trends where although the 12-week group logged in more often on average, the 4-week group spent more time on the project website. Participants in the 4-week group had a slightly higher self-rated engagement, and all of them recommended the program to others.

**Table 2:** Differences between 12- and 4-week formats (*t* tests).

Variables	12-week group, mean (SD)	4-week group, mean (SD)	*t* test (df)	*P* value
Time spent on the website (hours)	1.10 (1.30)	4.51 (5.68)	−1.65 (23)	.11
Number of log-ins	34.33 (59.00)	5.40 (5.98)	1.91 (23)	.07
Number of posts and messages	6.77 (6.49)	6.40 (6.25)	0.14 (23)	.89
Self-rated engagement	5.22 (2.48)	6.50 (2.41)	0.27 (23)	.22
Recommend program to others	0.89 (0.33)	1.00 (0.0)	0.03 (23)	.41

## Discussion

### Principal Findings

Youths with disabilities are a vulnerable population with an increased risk of social exclusion and, therefore, could benefit from mentoring [60]. Peer mentoring could help youths with disabilities to build social networks while improving academic and employment outcomes [10]. Our study addressed an important gap in the literature by exploring how mentors engage youths in an e-mentoring program while also comparing 2 different formats of the same intervention. Exploring this is important because a recent review highlighted that further work should explore what delivery formats work best [17,60].

Our findings reveal that mentors engaged youths in the e-mentoring program by providing informational, emotional, and tangible support. We noted more instances of mentors providing advice, empathy, and encouragement in the 12-week format compared with the 4-week format. We found fewer examples of providing advice, developing rapport, and social support from mentors in the 4-week format. It was interesting to note that we did not find significant differences between the 2 groups regarding the time spent in the Web forum, number of logins, number of posts, and self-rated engagement. The self-rated engagement of participants was lower than expected and lower than other mentoring studies; however, these studies used a different format (ie, Skype) [61]. The lower engagement in our study could be linked with the asynchronous nature of our forum and participants perhaps wanting more live interaction. Future studies should consider building in activities to help increase engagement [39,62].

Our results highlight that mentors experienced challenges with engaging participants, particularly in the 4-week format. These findings are consistent with other face-to-face and e-mentoring studies, showing that mentors had difficulties engaging youths and developing a rapport [47,62]. Other research similarly indicates that mentors play an essential role in engaging participants in a program [60]. Achieving successful outcomes through a mentoring relationship depends on the quality of the relationship [15]. Key components of peer mentor interventions include trained mentors, monitored implementation, structured activities, routine contact, and parental support [24,41,63,64]. A study by Cohen and Light [65] reports that the frequency and length of communications between mentors and mentees might be influenced by the availability of mentors and the quality of the match.

Our results revealed that the 12-week format seemed too long for mentors, whereas the 4-week format felt too rushed. Future studies should consider balancing mentors’ schedules along with the time it takes to develop a good rapport with mentees. The examples of advice provided by mentors in the 12-week format may have resulted from them having more time to elaborate on posts compared with the 4-week format where 3 topics were posted per week. Other research reports that the strength of communication between mentors can impact mentoring [37,60]. Indeed, it often takes time to develop a rapport with mentees.

Other e-mentoring studies reveal quite a range in the length of interventions from 4 to 24 weeks [66]. Both short- and long-term outcomes showed positive outcomes for youths with disabilities. It remains unclear which mentoring formats work best for youths with disabilities. Further work is needed to explore this in detail.

With regard to mediating factors and, specifically, gender of the mentors, our results indicated that our female mentor provided more emotional support than the male mentors, although one of the male mentors provided more informational support to mentees. This finding is consistent with a study from Allen and Eby [67], where female mentors were more likely to provide psychosocial support and males provided more career-related mentoring to mentees. These authors suggest that it is important to cultivate mentorship relationships with mentors of different genders to gain the most from mentoring [67], which supports our model of having a male and female mentor run the program together.

Other research indicates that in same-gender mentor relationships, the pairs are more likely to identify with each other, be more comfortable, and communicative [68]. For example, Ryan et al [69] found that in mentoring relationships with youths with developmental disabilities, the strongest connections included dyads where the mentee and peer mentor were the same gender. Ryan et al [69] also found that female mentees were more frequently in relationships with stronger connections as determined by mentor and mentee engagement and enthusiasm [69]. Further research should explore how a mentor’s gender might influence the mentee’s experience within a group-based environment.

### Limitations

It is important to highlight the limitations of our study. First, the data were drawn from a pilot feasibility study, which was collected from 1 site and had only 3 mentors and, therefore, may have limited generalizability. Second, there were several technical difficulties with the website over the course of the study (ie, difficulties logging in and glitches because of website upgrades) that may have impacted mentee engagement. Third, mentors may have felt rushed in responding to mentees, given their schedules and involvement in school and other activities. Fourth, there was staff turnover in mentors leading the discussion forum; however, they were provided the same training and had a similar level of experience; this could have affected outcomes. Fifth, we only had access to the total overall time that participants spent on the website and not daily or weekly averages. It would be important for future research to include this to test for any differences over time. Finally, there is a potential threat to validity, given that the 12-week program was delivered first followed by 2 implementations of the 4-week program. It is possible that mentors provided less information or shorter responses either because of boredom or forgetting their training.

### Future Directions

Future studies should consider exploring whether the timing of the year running the intervention (eg, summer months vs during the school year) affects the level of engagement in the mentoring program. It may be worthwhile for future research to compare and contrast peer versus professional mentors and explore any differences in the types of support provided by mentors. Other studies on e-mentoring for youths with disabilities involve email, virtual environments, *Skype* video calls, and phone calls [66]. A recent review of e-mentoring for youths with disabilities found that the majority of studies involved one-to-one mentoring and some had a combination of both one-to-one and group-based mentoring [66]. Thus, future studies should consider offering more than 1 approach to maximize youth engagement.

### Conclusions

Our study explored the role of mentors engaging youths with disabilities in an e-mentoring employment intervention. We also compared and contrasted mentors’ engagement strategies within a 12- and 4-week format. Our findings showed that mentors in the 12- and 4-week format engaged participants differently in providing informational and emotional support, although there were no differences in tangible support provided. Mentors reported the 12-week format was too long and lacked interaction between participants, whereas the 4-week format felt rushed and had fewer detailed responses from mentees. Further research should explore which mentoring formats work best for engaging youths with disabilities.

## Appendix

Multimedia Appendix 1 Format of 12- and 4-week mentoring intervention.

Multimedia Appendix 2 Mentor interview guide.

Multimedia Appendix 3 Types of mentor support within the discussion forum.

Multimedia Appendix 4 Mentor experience with the program.

## Abbreviations

e-mentor: electronic mentor
e-mentoring: electronic mentoring
