# Supplementary material for: A Web-Based Intervention for Youth With Physical Disabilities: Comparing the Role of Mentors in 12- and 4-Week Formats
Source: JMIR Pediatr Parent. 2020 Jan 8;3(1):e15813. doi: 10.2196/15813 (PMC6996779; doi:10.2196/15813)
Supplement: Multimedia Appendix 1 [file pediatrics_v3i1e15813_app1.docx]

Multimedia Appendix 2. Mentor interview guide.

1. How did you feel the mentoring program went?
2. How did you get along with your mentees?
3. What did you like best about the program?
4. What did you like least about the program?
5. How long did the calls with your mentee usually last? Was this too long, too short or just right?
6. On a scale of 0 to 10 where 0 is not at all engaged in the program and 10 is extremely engaged, how engaged did you feel the mentees were?
7. On a scale of 0 to 10 where 0 is not at all engaged in the program and 10 is extremely engaged, how engaged did you feel you were?
8. Were you in contact with other mentors throughout the program?
9. Do you feel you received adequate training and support to be an effective peer mentor?
10. What changes did you see in your mentors as a result of the calls?
11. What changes did you see in your self as a result of the mentoring experience?
12. Would you recommend the experience of being a peer mentor to another young adult with a disability?
13. Recruiting male mentees was a challenge for us. Do you have any thoughts on what could be changed in the program in order to make it more attractive to males?
14. Are you still in touch with any of your mentees?
15. Do you have any other comments that you would like to share with us about the program?
