# Supplementary material for: A Web-Based Intervention for Youth With Physical Disabilities: Comparing the Role of Mentors in 12- and 4-Week Formats
Source: JMIR Pediatr Parent. 2020 Jan 8;3(1):e15813. doi: 10.2196/15813 (PMC6996779; doi:10.2196/15813)
Supplement: Multimedia Appendix 3 [file pediatrics_v3i1e15813_app3.docx]

Multimedia Appendix 3. Types of mentor support within the discussion forum.

| Theme | Subthemes | 12-week format (group 1) | 4-week format (groups 2-3) | Representative Quotes |
| --- | --- | --- | --- | --- |
| Support | Informational | -Employment related (resume, looking for work, networking)  -Post-secondary  -Volunteering  -More instances of advice giving  -Posts were longer and  mentees wanted more advice | -Employment related (types of jobs, networking, accommodations)  -Posts were shorter  (less detail in examples) | ‘In the workplace, employers, especially corporations such as [media corporation], are required by law, to provide reasonable accommodations to persons with disabilities’ (Mentor 2, Group 1, 12-week)  ‘In terms of networking, it's when you connect with other professionals to gain knowledge and connections with others in a field you're interested in. Usually, people will talk about their own career aspirations and, if all goes well, the conversation can end with you exchanging contact information with the person you networked with.’ (Mentor 1, Group 3, 4-week)  “Hi Participant 105…As I was preparing to think about what I wanted to study in university, I remember doing a lot of research on the potential programs that were offered.  You'd mentioned that you are looking to apply to [University A] and [University B] so if you are still thinking about what program you are interested in pursuing, I'd suggest going onto the school websites and navigating them to see what they offer and what really stands out to you and go from there.” (Mentor 1, Group 1, 12-week) |
|  | Emotional | -Empathy, understanding  -Being vulnerable  -Female mentor offered more emotional support to mentees | -Offering encouragement  -Focused on solutions  -Female mentor offered more emotional support to mentees | ‘Don't be frustrated; You'll find something for sure, and at least you know what your limitations are so you know what to look out for/ask about next time you go for a job interview. Feel free to private message me if you'd like to chat about this further. (Mentor 1, Group 1, 12-week)  ‘Thank you so much for sharing your journey, it was very encouraging to read …having passion for what you're doing is a vital aspect of loving what you do, and since you seem to clearly have that, I'm sure you're going to excel in whatever path you choose to pursue!’ (Mentor 1, Group 2, 4-week). |
|  | Tangible | -No difference in strategy used  -Offering additional help  -Utilized only by Mentor 1 | -No difference in way strategy was used  -Offering additional help  -Utilized by Mentor 1 and Mentor 2 | ‘If you have any further questions about this, please don't hesitate to ask and we can work this out!’ (Mentor 1, Group 1, 12-week).  ‘In terms of whether you should finish high school or start your own business, it's great that you're looking to start your own business, however, my advice would be to complete high school first as this opens up so many options in terms of employment. Realistically, it's quite difficult to find a job if one doesn't have at least a high school diploma.’ (Mentor 1, Group 3, 4 week) |
