# Supplementary material for: A Web-Based Intervention for Youth With Physical Disabilities: Comparing the Role of Mentors in 12- and 4-Week Formats
Source: JMIR Pediatr Parent. 2020 Jan 8;3(1):e15813. doi: 10.2196/15813 (PMC6996779; doi:10.2196/15813)
Supplement: Multimedia Appendix 4 [file pediatrics_v3i1e15813_app4.docx]

Multimedia Appendix 4. Mentor experience with the program.

| Theme | Subthemes | 12-week format (group 1) | 4-week format (groups 2-3) | Representative Quotes |
| --- | --- | --- | --- | --- |
| Mentor experience | Lack of participation/ engagement among mentors | -Lack of interaction between mentees | -Lack of participation  -Short responses  -Lack of detailed posts | ‘the big issue was lack of responsiveness…I would have liked to see them interact with each other more. That would have been nice; but I think we did the best that we could’ (Mentor 1, Group 3 interview).  ‘The [posts] were too short definitely…no one was (really) wanting to continue conversations. You'd get an okay good first answer…but then whenever I put a response…they didn't open it up’ (Mentor 3, Group 3 interview). |
|  | Length / format of the study | -Slow pace  -Too long for mentees to handle  -Mentees found it too long (Mentee participation dropped in later weeks)  -Mentor engagement dropped | -Not enough time to elaborate  -Less interaction  -Felt rushed  -Did not get as close to mentees as 12-week group | ‘[The 4 week program] was better than the 12-week because the 12-week had a really slow pace, but there were some periods for example, when we had the short week (from a long weekend), we had a disadvantage’ (Mentor 2, Group 2 interview).  ‘I did not get as close to mentees as I would have liked to. Because I'm rushing through the topics each week…it doesn’t leave much time for a dialogue between me and my mentees’ (Mentor 1, Group 2 interview) |
